# Advanced Test Setup for Accelerated Aging of Plastics by Visible LED Radiation

**DOI:** 10.3390/ma13194261

**Published:** 2020-09-24

**Authors:** Moritz Hemmerich, Jörg Meyer, Frank Walther

**Affiliations:** 1 Photonics and Materials Science, Hamm-Lippstadt University of Applied Sciences, Marker Allee 76-78, 59063 Hamm, Germany; joerg.meyer@hshl.de; 2 L-LAB, Research Institute for Automotive Lighting and Mechatronics, Rixbecker Str. 75, 59552 Lippstadt, Germany; 3 Department of Materials Test Engineering, TU Dortmund University, Baroper Str. 303, 44227 Dortmund, Germany; frank.walther@tu-dortmund.de

**Keywords:** advanced irradiation setup, photodegradation, blue light, LED, polycarbonate, accelerated aging, optical materials

## Abstract

In this article, a newly developed test setup for the aging of optical plastics by visible radiation (450 nm) is presented. In addition to a comprehensive monitoring of the operating parameters and an efficient cooling of the high-power multiple chips on board the LEDs used, the plastic samples can be fully temperature-controlled, independent of the radiant power of the LED, due to fluid driven thermostatization. The sample surface temperatures and irradiance values were verified by in situ measurements and simulations. To validate the test setup, polycarbonate samples with well-known aging behavior were aged for 1896 h. By spectroscopic IR and UV/vis analysis of the samples at different aging times, known optical aging results of polycarbonate could be observed, which proves the intended operationality of the system.

## 1. Introduction

The market share of LEDs in lighting applications has increased rapidly. In 2014, LEDs made up less than 4% of the total lumen-hours in lighting. For 2023, a percentage of around 84% is predicted [1]. The advantages of LEDs over conventional light sources are their long operational lifetime (more than 15,000 h on average), high luminous efficacy, and the possibility of compact design and simple installation [2,3,4,5]. However, this poses problems for the stability of the optical plastic components, such as lenses, cover lenses, or housing, which are installed close to the LED. Two of the most commonly used transparent plastics in the field of lighting applications are polycarbonate (PC) and poly(methyl methacrylate) (PMMA) [6]. Due to ever more compact designs, such as high-resolution LED matrix headlights [7] or light guides with very small light-emitting surfaces, plastic components associated with LED lighting are exposed to increasing levels of visible radiation. The coating of a blue emitting diode chip with a luminescent color converter is the most commonly used industrial technology to produce a white LED [8,9]. Even after partial down-conversion, these LEDs still contain a high proportion of high-energy blue radiation that can drive the degradation of plastics. To better assess and more accurately predict the stability of optical plastics in LED applications, it is crucial to develop a suitable test setup, which gives valid and reproducible accelerated results on the aging behavior. Since real-time aging takes too long, the common procedure is to expose the samples to irradiation that is several times higher than in reality. This allows the degradation behavior to be simulated on suitable time scales and thus to be predicted. [10] The degradation behavior of various plastics, e.g., PC [11,12,13,14], PMMA [15], and PE [16], under UV-irradiation has been investigated in several studies. In these investigations, mostly commercial aging setups based on xenon arc lamps manufactured by ATLAS^TM^ were used. Both purely thermal- [17,18,19] and photodegradation by blue light [20,21,22,23,24,25,26] of bispenol A PC were investigated by Mehr et al. For the experiments, a self-made apparatus was used, in which samples are heated simultaneously by a hot plate and irradiated by several LEDs. Sikora et al. investigated the effects of pulsed UV and white light irradiation on PMMA. For this purpose, a setup was used in which a single sample was irradiated at several locations with different wavelengths and pulse widths [15]. Gandhi et al. also investigated the accelerated aging of PC by a blue emitting LED, whereby the focus of the work was on the determination of acceleration parameters in order to be able to make service life predictions [27]. The experiments were performed in a commercial test device called “Elevated Temperature Irradiance Chamber” (ETIC), in which the samples are irradiated via a lightguide in a fan temperature-controlled test chamber. To the best of our knowledge, this test equipment is no longer commercially available.

All these investigations provided interesting findings to better understand and predict the aging behavior of PC and PMMA. To be able to carry out further and more in-depth aging studies on different optical plastics in the future, we developed a new test setup termed “Monitored Liquid Thermostatted Irradiation Setup” (MLTIS) for accelerated aging with blue light. This test setup has been designed considering experience with the test setups presented above and has been improved with regard to accuracy, handling, and scalability.

The most important features of the MLTIS are briefly explained below:

- The sample chamber is thermostatted by the circulation of a liquid, which enables a temperature control of the sample independent of the radiant power of the LED.
- All operating parameters of the experimental setup are measured or simulated in advance.
- The specimens are aged separately from each other in individual chambers, which avoids mutual influence of the samples during aging.
- The chamber geometry can theoretically be replaced at will by other geometries, so that samples of different shapes and sizes can be tested.
- The LED and sample temperature as well as the irradiation on the sample surface are monitored during the entire test duration.
- An independent design of the MLTIS enables massive parallelization by using further chambers.

A detailed description of the MLTIS’ design and functionality is given in the next section. To demonstrate the functionality of the described test setup, first aging experiments were carried out using exemplary PC samples, due to the well-described aging phenomena that occur with such samples. The samples were exposed to both optical and thermal stress to induce photo-thermal degradation effects. The PC samples were analyzed by ATR-FTIR and UV/vis spectroscopy.

## 2. Materials and Methods

### 2.1. Experimental Setup

#### 2.1.1. Design of the “Monitored Liquid Thermostatted Irradiation Setup” (MLTIS)

**Figure 1:**
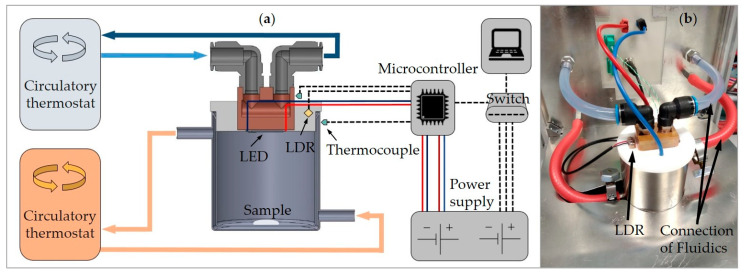
Design of the MLTIS: (**a**) schematic representation; and (**b**) photo of an aging unit with corresponding sensors, electrical, and fluidic connections. The two green thermocouples are mounted on the backside of the copper block and the sample chamber.

The basic design of the MLTIS is shown schematically in Figure 1a. The central part of the MLTIS is the double-walled stainless-steel sample chamber. The chamber is continuously thermostatted by the circulation of a liquid. By suitable selection of the liquid, the used circulating thermostat can realize temperatures ranging from −10 to 140 °C, whereby the upper temperature limit is currently 120 °C due to the gaskets used and the liquid medium, viz. water.

The high-power LED used for aging is positioned above the sample. To ensure a long service lifetime and high efficiency, the LED is mounted heat-conductingly on a copper block, which is water-cooled by a further circulating thermostat. The water temperature is 34 °C, which ensures a constant cathode temperature of the LED of 50 °C throughout the experiment’s duration. The copper block, in turn, is embedded in a PTFE cover which is placed on the sample vessel. Each sample chamber is equipped with three sensors: two thermocouples (type-K, RS Components, Frankfurt am Main, Germany) and one photo resistor (hereafter abbreviated as LDR, LDR-KY-018, Joy-IT, Neukirchen-Vluyn, Germany). The first thermocouple measures the temperature of the outer wall of the sample chamber, which corresponds by a rough approximation to the temperature of the sample. The second thermocouple is located on the copper block for an indirect measurement of the cathode temperature of the LED. The LDR is embedded in an indentation in the PTFE cover and is used for indirect determination of the irradiance on the bottom of the sample chamber (Figure 1b). The sensor data of each sample chamber are sent separately to a microcontroller for further processing. Afterwards, the data from each sample chamber are transmitted from an attached Ethernet board via a local network to a central processing unit. The power supply for the LED is provided by a 750 W constant current power supply (Genesys 750W, TDK-Lambda, Tokyo, Japan) whose operating parameters are also sent to the central computing unit via Ethernet. The three sensor values of all running sample chambers are retrieved sequentially via the corresponding IP addresses by a MATLAB (R2018b) script. The query time can be chosen as desired.

**Figure 2:**
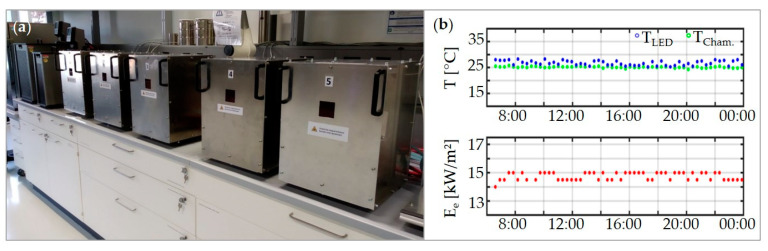
(**a**) Photograph of the enclosures for radiation-protection; and (**b**) exemplary presentation of the MATLAB Plot-Window, showing the temperatures of sample chamber and copper block (top) and the calculated irradiance (from LDR-readouts) on the bottom of the sample chamber (bottom) over a time period of 18 h.

By monitoring (Figure 2b) and recording all operating conditions, a controlled and traceable aging can be ensured for each sample chamber individually. In the case of irregularities, an error log is created, and the system of the corresponding sample chamber is shut down. To achieve a higher throughput and better comparability of the experiments, twelve sample chambers can currently be operated simultaneously. As shown in Figure 2a, the reactors are operated in radiation-proof enclosures for eye-protection. For inspection during the experiment, the sample chambers can be observed through a small window, which strongly attenuates the blue radiation. The upper part of the enclosure can be removed for sample handling and experiment setup.

#### 2.1.2. Sample Chamber

The purpose-built double-walled cylindrical stainless-steel sample chamber described in this work has an inner diameter of 68 mm and a height of 80 mm. The inner side of the stainless-steel chamber has a reflectivity of 51.9% at 450 nm. The LED is located at a distance of 65 mm above the sample. A further sample chamber with a diameter of 100 mm and a height of 156 mm (TSS-G 1000 W, KGW-Isotherm, Karlsruhe, Germany) is available for the aging of larger samples. Further sample chamber geometries can be realized if required.

#### 2.1.3. Multiple Chips on Board LED

Blue high-power multiple chips on board (MCOB) LEDs (CLU048-1818C4-B455-XX, Citizen Electronics, Kamikurechi, Japan) were used to age the samples. The LEDs have a narrow band spectrum with a full width at half maximum (FWHM) of 17 nm and a peak wavelength of 450 nm. The maximum electrical power consumption is 202 W. The special feature of the LED is its long service life, which has been proven in real-time tests by the manufacturer. To determine the spectral power distribution, the LED module was measured at a constant cathode temperature of 50 °C in an integrating sphere (IS 3900, Optronic Laboratories, Orlando, FL, USA). The current was varied in 10% steps from 0.486 (30%) to 3.24 A (200%), referring to the typical current of 1.62 A (100%), given in the data sheet. To increase the service life, the LED is normally operated at 1.134 A (70%) during aging tests.

#### 2.1.4. Irradiance Simulation

**Figure 3:**
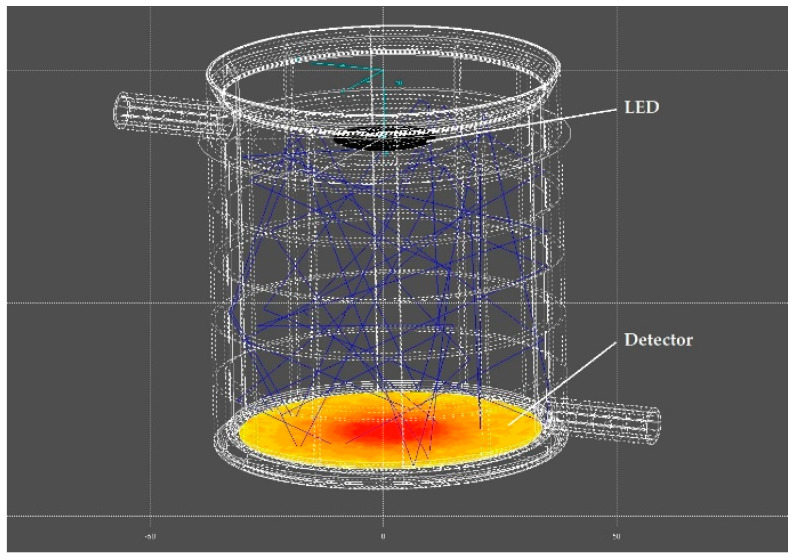
Representation of the raytracing setup with a beam reflected 50 times. The detector visualizes the irradiance in false colors.

No equipment for optical measurements was available to determine the irradiance at the bottom of the sample chamber, due to the high optical power of the LED which would saturate or even destroy unfit sensors (e.g., those housed in colored plastics). For this reason, a photometric raytracing simulation was used. The optical parameters of the LED, the optical properties of all irradiated materials, and the geometry were determined. The optical parameters of the LED were taken from the integration sphere measurements as described above. The reflectivity of the stainless steel and PTFE parts, which are exposed to the radiation, were determined using a smaller integration sphere (ISR-2600, Shimadzu, Kyoto, Japan). The geometry was represented as a CAD model. The simulation was carried out with an in-house raytracing simulation software for optical applications. Figure 3 shows the simulation setup using a ray that is reflected 50 times. After 50 reflections, the simulation was interrupted for the respective ray, since the remaining energy is less than 10 ppt. In total, each simulation was performed with 5 million rays.

#### 2.1.5. Sample Temperature Measurement

To ensure that the sample temperature can be controlled independently of the radiant power of the LED, the sample temperatures have been determined as a function of the set temperature of the thermostat. A direct measurement of the sample temperature with a thermocouple, with the LED driven at the desired 70% nominal current, was not expedient, because the thermocouple absorbs a high amount of radiation and therefore does not provide reliable temperature values for transparent samples. For this reason, an infrared (IR) camera (VarioCAM HR, Infratec, Dresden, Germany) was positioned statically above the sample chamber. After a period of 30 min, during which temperature equilibration was achieved, the LED was switched off, the cover of the chamber was removed, and a thermal image of the PC sample surface was taken immediately. The temperature values of three independent measurements of the sample surface were averaged afterwards. This process was performed for nominal temperatures of 10–90 °C in 10 °C increments.

### 2.2. Aging and Analysis Methods

#### 2.2.1. Sample Geometry and Aging Conditions

Polycarbonate granulate (Tarflon LC 1500, Idemitsu Kosan, Tokyo, Japan) was injection molded into disks with a diameter of 20 mm and a height of 1.5 mm. Injection molding was carried out with a lab scale instrument (Minijet Pro, Thermo Fisher Scientific, Waltham, MA, USA). Cylinder temperature, mold temperature, and injection and post pressure were varied in advance to achieve the highest possible transparency of the samples in the wavelength range from 300 to 800 nm. The best results were achieved at a cylinder temperature of 85 °C, a mold temperature of 255 °C, an injection pressure of 800 bar, and a post pressure of 560 bar. Seventeen sample disks, produced using these parameters, were selected for aging. Due to the high number of samples, the effects of measurement or material errors for individual measurements or samples can be minimized.

Since only two sample chambers were available at the start of the experimental series, one sample each was positioned centrally in the chamber. The remaining samples were positioned around them. Additionally, to speed up the aging process, the distance between the samples and the LED was reduced to 18 mm. The maximum irradiance *E*_2_ at any distance *d*_2_ from the LED can be approximated as a function of a known irradiance *E*_1_ at a certain distance *d*_1_, according to the photometric distance law [5] (Equation (1)).

(1) $$E_{2}\;=\;\frac{E_{1}\cdot d_{1}^{2}}{d_{2}^{2}}$$

Depending on the calculated maximum irradiance in the center, the irradiance drop to the peripheral areas *E*(α) can be estimated as a function of the angle α, between the center and an arbitrary point, according to the cos^4^ -Law (Equation (2)).

(2) $$E(\alpha )\;=\;E_{2}\cdot \;\cos^{4}(\alpha )$$

The samples at the edges were aged for 1896 h in total. UV/vis and FTIR analyses were carried out both before aging and after each of 24 successive aging periods.

#### 2.2.2. FTIR-Spectroscopy

Infrared spectra were obtained with the infrared spectrometer (Nicolet iS50, Thermo Fisher Scientific, Waltham, MA, USA) in the mid-IR range from 4000 to 400 cm^−1^ in attenuated total reflection mode (ATR). Twenty scans were averaged at a resolution of 4 cm^−1^. For better comparability of the spectra recorded at different aging times, the spectra of all samples were normalized at 1014 cm^−1^ by a custom LabTalk algorithm, since no changes occur at this wavenumber. In addition, the spectra were baseline corrected at 23 positions. The exact positions of the baseline corrections are shown in Table A1 of the Appendix. To ensure that measurements are always taken at the same point on the sample, a dedicated 3D-printed sample-holder was used, which is placed on the measuring surface of the infrared spectrometer and ensures that the samples are in the same position for each measurement.

#### 2.2.3. UV/vis-Spectroscopy

UV/vis measurements were performed with the UV/vis spectrometer (UV-2600, Shimadzu, Kyoto, Japan). The transmission of the aged samples at different times was performed in the range of 200–800 nm with a resolution of 1 nm, at medium scan speed. An integrating sphere (ISR-2600, Shimadzu, Kyoto, Japan) was attached to the spectrometer to determine reflectivity where needed. From the transmission spectra of the aged samples, a calculation according to Equation (3), as defined by DIN 6167 [28], was performed to determine the yellowness index (*YI*) for a standard 2° observer and standard illuminant C.

(3) $$YI\;=\;100\;\times \;\frac{1.277\;\cdot \;X\;-\;1.059\;\cdot \;Z}{Y}$$

where *X*, *Y*, and *Z* are the tristimulus values of the samples.

## 3. Results

### 3.1. Characterization of the Experimental Setup

#### 3.1.1. LED Radiant Flux and Spectrum

**Figure 4:**
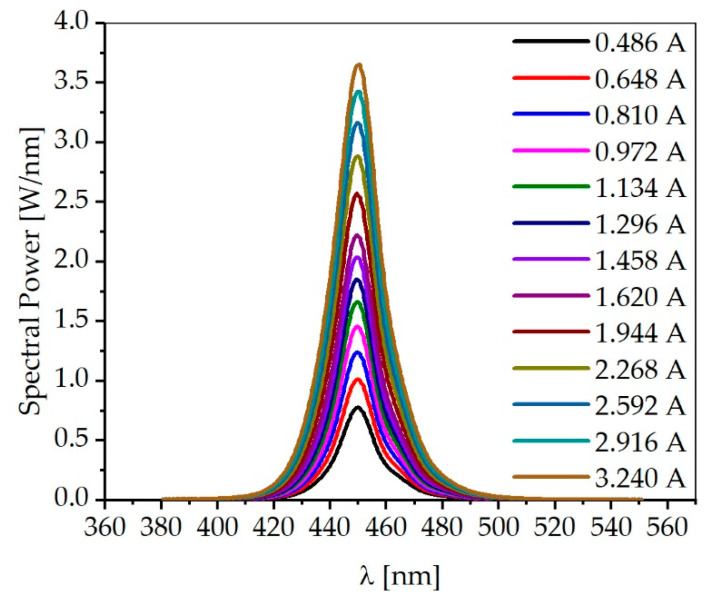
Spectra of the blue high-power LED used for different driving currents between 0.486 and 3.240 A.

**Figure 5:**
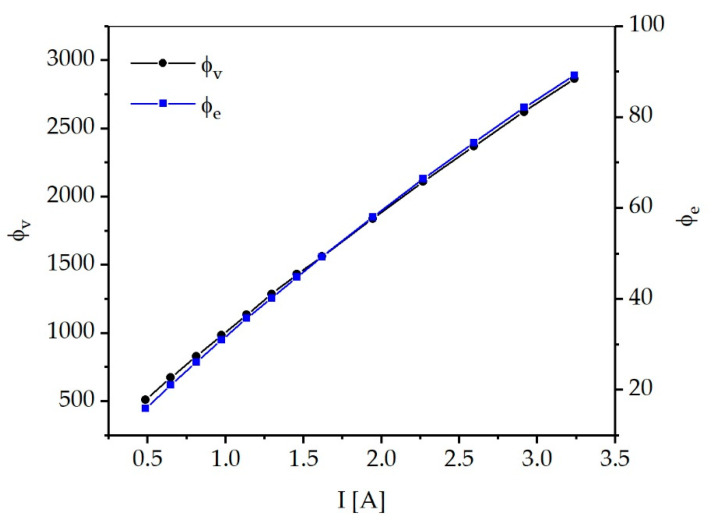
Radiant (ф_e_) and luminous flux (ф_v_) values for different currents between 0.486 and 3.240 A.

Figure 4 shows the spectrum of the LED used at currents ranging from 0.486 to 3.24 A. At the currents measured, the FWHM ranges between 15.4 and 19.4 nm. At a current of 1.134 A, the FWHM is 16.3 nm. The peak wavelength is constant at 450 nm. The radiant flux ф_e_ values calculated by integrating the spectra are shown in Figure 5. In addition, the corresponding luminous flux ф_v_ values are shown. An approximately linear relationship can be observed between the current and the radiant flux as well as the luminous flux. A radiant flux of 35.79 W is obtained for the experimentally used operating conditions of 1.134 A and 50.9 V.

**Figure A1:**
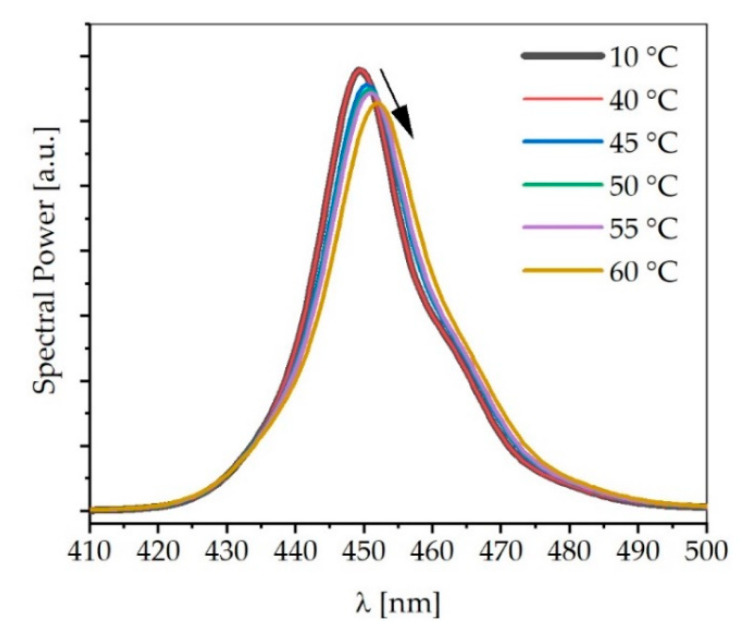
Plot of the LED spectrum as a function of the LED-thermostat temperature.

This corresponds to an efficiency of 62% for the conversion of electrical power into optical power, which indicates an efficient mode of operation of the LED. Table 1 shows the remaining radiant flux and luminous flux values as a function of the current. It can be observed that the efficiency decreases with increasing current. Therefore, the preferred operation at 70% of the electrical power constitutes a good compromise between high optical power and economical operation of the LED. Figure A1 in the Appendix shows the spectrum of the LED depending on the set temperature of the thermostat used for LED cooling.

#### 3.1.2. Degree of Irradiance at the Sample Level

**Figure 6:**
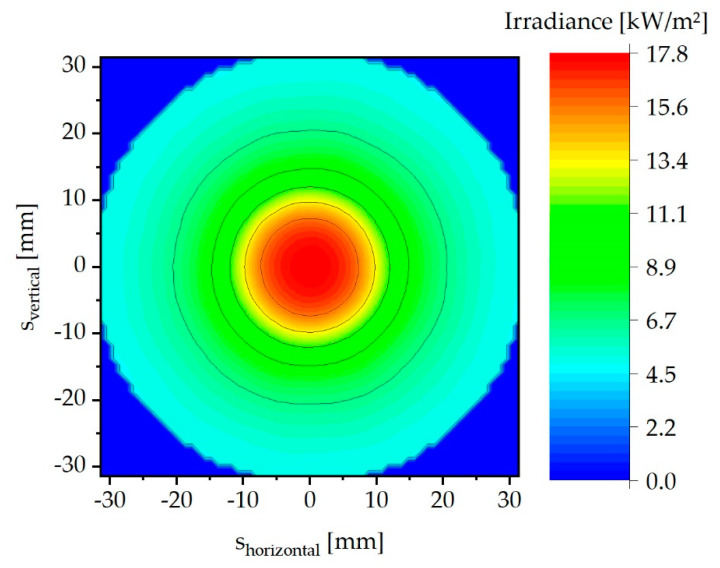
Exemplary irradiance distribution on the sample chamber ground in false colors at a current of 1.296 A.

As mentioned above, the irradiance on the bottom of the sample chamber was simulated by photometric raytracing. Figure 6 shows an exemplary result of a simulation in which the position-dependent irradiance level is displayed in false colors. The highest irradiance results in the center. From there outward, the irradiance decreases. The total irradiance is radially symmetrically distributed.

**Figure 7:**
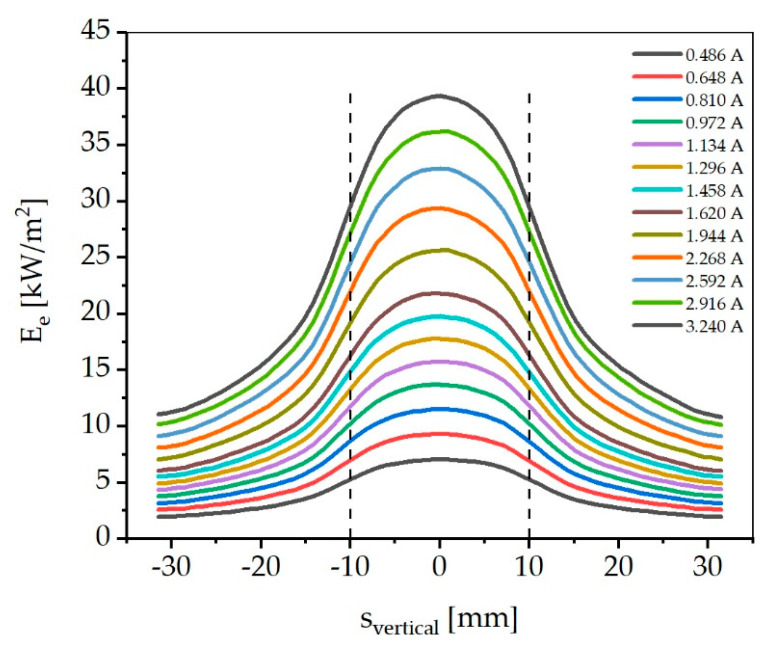
Vertical sections through the irradiance distribution on the virtual detector of the raytracing simulation. The area between the dashed lines marks the position of a centered sample.

For better illustration, the resulting irradiances for different currents are shown in vertical cross-sections in Figure 7. The area marked by the dashed lines has a size of 20 mm and thus indicates the area in which the round sample disk would be located. In the marked area, 51% of the total optical power is incident. In relation to the total irradiance, the decrease in the marked area is about 35%.

**Figure 8:**
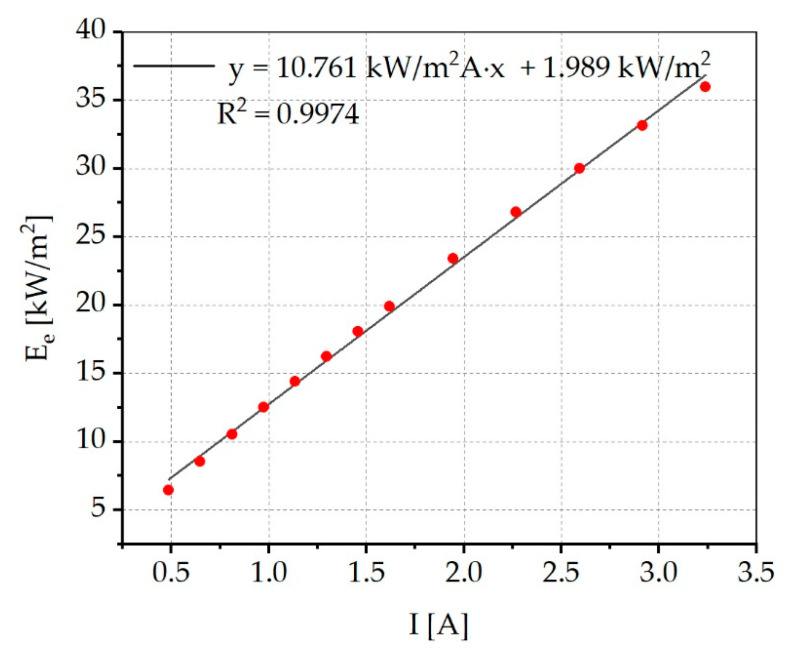
Mean irradiance in the sample area for different currents. The irradiance increases by about 10.7 kW/m^2^ per ampere.

Figure 8 shows the mean irradiance in the sample area for different operating currents. The fitting function used shows a linear relationship between the current and the mean irradiance, whereby the irradiance increases by about 10.7 kW/m^2^ per ampere. The typical current of 1.134 A results in an average irradiance of 14.4 kW/m^2^. We are aware of the fact that the presented graph in Figure 8 must typically pass through the origin and that there will be a flattening of the curve for larger currents. However, to fit the data between the highest and lowest measured value, a linear function with a positive y-axis section was deliberately chosen, as this approximates the existing data points in an uncomplicated and very accurate way. This allows irradiances to be calculated precisely within the available current range.

#### 3.1.3. Sample Temperature

**Figure 9:**
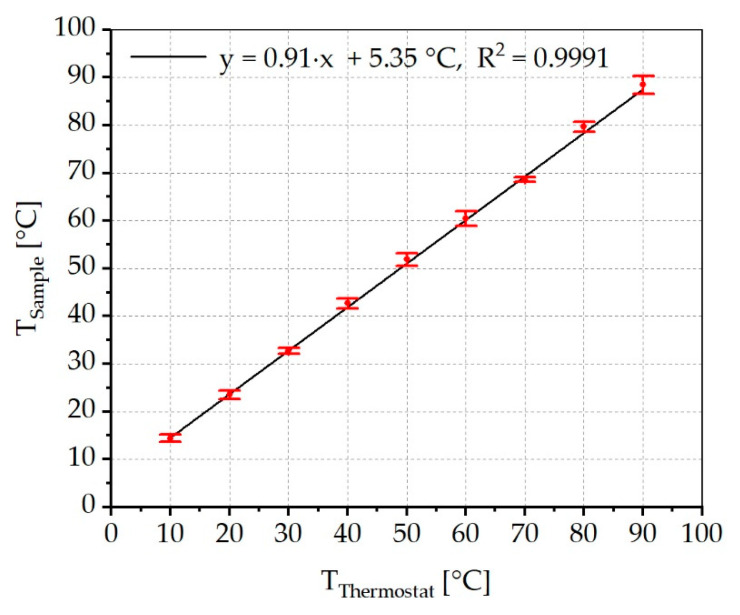
Sample surface temperature as function of the set thermostat temperature. The sample surface temperature increases by 0.91 °C per 1 °C of the set thermostat temperature.

Figure 9 shows the results of the sample surface temperature measurement using an IR camera. These measurements were carried out at the typical current of 1.134 A and a resulting irradiance of 14.4 kW/m^2^. The results indicate that, for the measured temperature range, any sample temperature can be achieved, independent of the radiant power, if the samples do not interact strongly with the impinging radiation (i.e., optically transparent samples). Due to the linear relationship between the temperatures set at the thermostat and the sample surface temperature, it can be assumed that sample temperatures <10 °C and >90 °C can be achieved. The thermostat temperature required to reach a selected sample surface temperature can be determined in advance based on the measurement data and the fitting function of these data. Basically, the sample surface temperature increases by 0.91 °C when the thermostat temperature is increased by 1 °C. By increasing the irradiance, either by increasing the current or by decreasing the distance between the LED and the sample, the sample temperature, at the same thermostat temperature, will also increase. It can be assumed that the function will remain linear due to the efficient temperature control.

### 3.2. Polycarbonate Aging Results

Based on a maximum irradiance of 15.7 kW/m^2^ at a distance of 65 mm between the LED and the bottom of the chamber, equation 1 provides a maximum irradiance of 205.2 kW/m^2^ at the center, for a distance of 18 mm between sample and LED. According to Equation (2) and measurements by the IR camera, the samples placed in the center are exposed to an average irradiance of 168.6 kW/m^2^ and an average temperature of 104 °C. The samples at the rim are exposed to an average irradiance of 69.0 kW/m^2^ and an average temperature of 62 °C.

**Figure 10:**
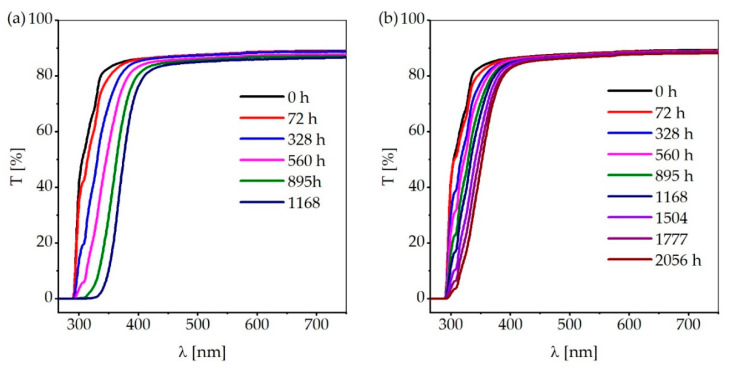
Mean value of the transmission spectra of PC samples at different aging times: (**a**) samples stored at the center of the test chamber; and (**b**) samples stored at the rim of the test chamber.

Figure 10 displays the mean value of the transmission spectra of PC samples at the center (Figure 10a) and at the rim (Figure 10b) at different aging times. It can be noticed that the decrease of transmission starts at wavelengths in the UV range. This initial decrease in transmission is due to the growth of new absorption bands in the range of 320–355 nm as a result of photodegradation. These absorption bands are ascribed to the photo-Fries rearrangement products L1 and L2 [11,13,14]. With prolonged aging, the decrease in transmission shifts into the visible wavelength range, which increases the absorbed amount of blue radiation by the samples, resulting in a yet stronger decrease in transmission. From this point on, the degradation of the plastic will be self-enhancing.

**Figure 11:**
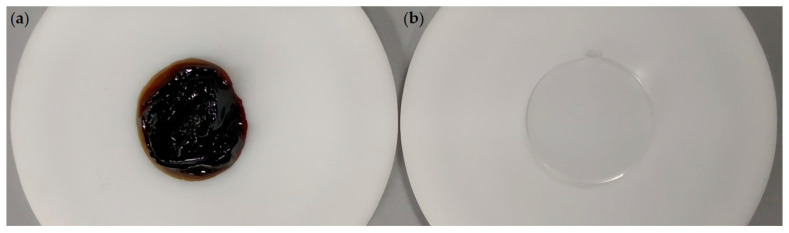
(**a**) PC sample stored in the center after complete failure after 987 h; and (**b**) unaged PC sample.

Within a few hours after the measurement at 987 h, the samples stored in the center became completely black and decomposed. Figure 11 shows the mentioned sample (Figure 11a) and an PC sample before aging (Figure 11b). This sudden complete failure of the plastic can be caused by the self-amplifying effect described above and by impurities within the samples that act as a trigger or accelerator [10,29]. The process reveals that, once originally transparent samples start to discolor strongly, the originally selected sample temperature can no longer be ensured.

**Figure 12:**
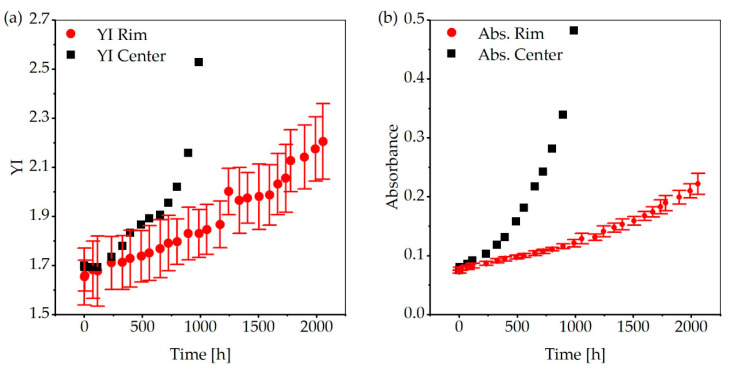
Progression of the *YI* and the absorbance at 360 nm with increasing aging time: (**a**) *YI*; and (**b**) absorbance at 360 nm. Due to the limited number of samples, no measurement accuracy could be determined for the samples in the center.

To compare the samples placed in the center and at the rim, the *YI* calculated according to Equation (3) is shown in Figure 12a. The representation reveals that the deviation of the *YI* between the individual samples is relatively high. Since, according to Gandhi et al. [27], there is a close relationship between the *YI* and the absorbance at 360 nm, Figure 12b also shows the development of the absorbance at 360 nm with increasing aging time for both sample types. The progressions of the absorbance at 360 nm and the *YI* show a similar behavior, whereby the measuring accuracy of the absorbance at 360 nm is considerably higher. Even if the limited number of samples stored in the center of the chamber does not allow a statement about the uncertainty of the measurement, the samples in the middle show a significantly stronger exponential growth of both the YI and the absorbance at 360 nm, compared to the samples at the edges. This verifies the mentioned self-amplifying effect of optical aging. To detect molecular changes on the surface of the PC samples as a result of photodegradation, FTIR was performed in ATR mode. The scan results of all samples (12 samples from the rim and 2 from the center) at all sampling times were averaged, normalized, and base line corrected according to the described algorithm. The IR spectra of the samples placed in the center and at the rim do not differ in quality. However, as expected, the changes in the samples stored in the center are more pronounced after a shorter time. Since more individual IR measurements were performed on the samples from the rim and the measurements have a higher statistical accuracy, due to the higher number of samples, these measurements are presented below.

**Figure 13:**
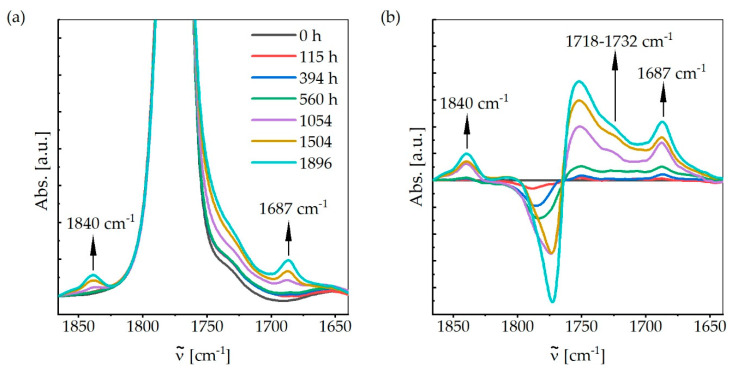
IR-spectra from 1640 to 1890 cm^−1^ after different aging times: (**a**) carbonyl region from 1640 to 1890 cm^−^^1^; and (**b**) IR-spectra compared to the spectrum of the unaged samples.

Figure 13 illustrates the IR-spectra of the carbonyl region from 1640 to 1890 cm^−1^ after different aging times. Outside this area, no clear changes could be observed. With increasing aging time, the increase of two distinct peaks at 1840 and 1687 cm^−1^ can be observed. For better illustration, Figure 13b shows the changes in the IR-spectra compared to the spectrum of the unaged samples. From this representation, a further increase in absorbance between 1718 and 1732 cm^−1^ can be identified. Furthermore, the carbonyl-band at 1772 cm^−1^ shifts to higher wavenumbers.

The increase in absorbance at 1687 cm^−1^ can be attributed to the photo-Fries rearrangement product L1 mentioned above. An increase of the photo-Fries rearrangement product L2 at 1619 cm^−^^1^ could not be detected clearly. The absorption bands at 1840 and between 1718 and 1732 cm^−^^1^ indicate the formation of aliphatic chain acid and cyclic anhydride. These products indicate photooxidative processes [11,13,14].

## 4. Discussion and Outlook

The newly developed “Monitored Liquid Thermostatted Irradiation Setup” (MLTIS) test setup provides a number of advantages over existing setups for the accelerated aging of optical materials with blue light. Samples can be aged in a wide temperature range independent of the LED power. This offers the possibility to carry out further aging experiments, e.g., at room temperature, allowing a distinction between the influence of thermal- and photodegradation. Furthermore, relevant operating parameters are monitored during the entire experiment to ensure traceable results. All relevant optical parameters of the setup were measured in advance. To determine the irradiance, a raytracing simulation was carried out using these data. Due to a flexible and modular design, samples in different geometries can be tested, while up to twelve sample chambers can be operated simultaneously and independent of each other.

To validate the test setup, polycarbonate samples with known aging behavior were aged by the MLTIS. Samples placed on the rim of the sample chamber were aged at a temperature of 62 °C (104 °C for samples in the center) and an irradiance of 69.0 kW/m^2^ (168.6 kW/m^2^ for samples in the center) for a total of 1896 h. Based on the parameters, it can be assumed that the samples are not only undergoing a photodegradation due to the high irradiation but also a thermal degradation even at the lower temperatures. UV/vis spectroscopy showed a decrease in transmission of the samples in the short wavelength range of 320–355 nm, with increasing aging time. This decrease is attributed to known absorption bands of photo-Fries rearrangement products. Additionally, a correlation between the *YI* and the absorption at 360 nm could be shown. By IR spectroscopy, new absorption peaks at 1690, 1840, and between 1718 and 1732 cm^−^^1^ could be observed. These peaks can be assigned to the photo-Fries rearrangement product L1, as well as to the formation of aliphatic chain acid and cyclic anhydride, which indicate photooxidative processes. These results are well in agreement with known results for the photo-thermal degradation of polycarbonates, confirming the validity of the MLTIS. In summary, the novel design of this apparatus allows for reliable temperature variable testing of materials, with regard to their resistance to optical radiation. To implement a holistic and sustainable development in the field of lighting, it is important to investigate alternatives to the established optical plastics in the future. One possible alternative is the biodegradable bioplastic polylactide (PLA), which is based exclusively on renewable raw materials and has excellent optical properties in its amorphous state [30]. In the future, in-depth investigations on the resistance of PLA (e.g., in comparison to PC) to optical radiation will be carried out. In particular, due to the temperature control capabilities of the MLTIS, the PLA samples can be kept at room temperature to avoid undesirable crystallization effects during the aging process.

## Figures and Tables

**Table 1:** Luminous flux, radiant flux, and efficiency of the high-power LED used as a function of the current.

Current I (A)	Luminous Flux ф_v_ (lm)	Radiant Flux ф_e_ (W)	Efficiency η (%)
0.486	512	15.96	67.3
0.648	674	21.09	65.9
0.810	831	26.17	64.7
0.972	983	30.98	63.2
1.134	1135	35.79	62.0
1.296	1287	40.24	60.5
1.458	1431	44.84	59.4
1.620	1563	49.27	58.3
1.944	1841	58.05	56.3
2.268	2110	66.48	54.5
2.592	2370	74.42	52.7
2.916	2624	82.15	51.1
3.240	2866	89.25	49.5

## Appendix A

To determine a suitable temperature for the LED cooling, the spectrum of the LED was measured by varying the thermostat temperature. The results are illustrated in Figure A1. It can be observed that, up to the temperature of 40 °C, the LED emits a constant spectrum with a peak wavelength of 450 nm. From 45 °C, the spectrum shifts to longer wavelengths and the maximum spectral power decreases. Therefore, to ensure that the emitted spectrum of the LED remains constant during the test period, the thermostat temperature of 34 °C was selected.

Table A1 shows the exact wavenumbers for the baseline correction of the recorded FTIR spectra. At these wavenumbers, the absorbance of all spectra at a certain measurement time are forced to zero.

**Table A1:** Exact wavenumbers for the baseline correction.

Baseline Correction Number	Wavenumber (cm^−1^)
1	599.763
2	689.920
3	794.059
4	865.413
5	951.714
6	1050.55
7	1345.128
8	1432.392
9	1541.352
10	1569.315
11	1621.385
12	1866.786
13	1925.123
14	1987.318
15	2155.097
16	2268.396
17	2427.015
18	2670.006
19	3005.083
20	3266.394
21	3488.171
22	3717.180
23	4000.188
